# Predicting Modified Fournier Index by Using Artificial Neural Network in Central Europe

**DOI:** 10.3390/ijerph191710653

**Published:** 2022-08-26

**Authors:** Endre Harsányi, Bashar Bashir, Firas Alsilibe, Muhammad Farhan Ul Moazzam, Tamás Ratonyi, Abdullah Alsalman, Adrienn Széles, Aniko Nyeki, István Takács, Safwan Mohammed

**Affiliations:** 1 Institute of Land Use, Technical and Precision Technology, Faculty of Agricultural and Food Sciences and Environmental Management, University of Debrecen, 4032 Debrecen, Hungary; 2 Institutes for Agricultural Research and Educational Farm, University of Debrecen, Böszörményi 138, 4032 Debrecen, Hungary; 3 Department of Civil Engineering, College of Engineering, King Saud University, P.O. Box 800, Riyadh 11421, Saudi Arabia; 4 Department of Transport Infrastructure and Water Resources Engineering, Széchenyi István University, Egyetem tér 1, 9026 Gyor, Hungary; 5 Department of Civil Engineering, College of Ocean Science, Jeju National University, 102 Jejudaehakro, Jeju 63243, Korea; 6 Department of Biosystems and Food Engineering, Faculty of Agricultural and Food Sciences, Széchenyi István University, Vár Square 2, 9200 Mosonmagyarovar, Hungary; 7 Doctoral School of Humanities, University of Debrecen, Egyetem Tér 1, 4032 Debrecen, Hungary

**Keywords:** land degradation, machine learning, climate change, Hungary

## Abstract

The Modified Fournier Index (*MFI*) is one of the indices that can assess the erosivity of rainfall. However, the implementation of the artificial neural network (ANN) for the prediction of the *MFI* is still rare. In this research, climate data (monthly and yearly precipitation (*p_i_*, *P_total_*) (mm), daily maximum precipitation (*P_d-max_*) (mm), monthly mean temperature (*T_avg_*) (°C), daily maximum mean temperature (*T_d-max_*) (°C), and daily minimum mean temperature (*T_d-min_*) (°C)) were collected from three stations in Hungary (Budapest, Debrecen, and Pécs) between 1901 and 2020. The *MFI* was calculated, and then, the performance of two ANNs (multilayer perceptron (MLP) and radial basis function (RBF)) in predicting the *MFI* was evaluated under four scenarios. The average *MFI* values were between 66.30 ± 15.40 (low erosivity) in Debrecen and 75.39 ± 15.39 (low erosivity) in Pecs. The prediction of the *MFI* by using MLP was good (*NSE*_Budapest(SC3)_ = 0.71, *NSE*_Pécs(SC2)_ = 0.69). Additionally, the performance of RBF was accurate (*NSE*_Debrecen(SC4)_ = 0.68, *NSE*_Pécs(SC3)_ = 0.73). However, the correlation coefficient between the observed *MFI* and the predicted one ranged between 0.83 (Budapest (SC2-MLP)) and 0.86 (Pécs (SC3-RBF)). Interestingly, the statistical analyses promoted SC2 (*P_d-max_* + *p_i_ + P_total_*) and SC4 *(P_total_* + *T_avg_* + *T_d-max_* + *T_d-min_)* as the best scenarios for predicting *MFI* by using the ANN–MLP and ANN–RBF, respectively. However, the sensitivity analysis highlighted that *P_total_*, *p_i_*, and *T_d-min_* had the highest relative importance in the prediction process. The output of this research promoted the ANN (MLP and RBF) as an effective tool for predicting rainfall erosivity in Central Europe.

## 1. Introduction

In many regions across the world, the most predominant type of land degradation is soil erosion, which has adverse environmental and socioeconomic consequences [1,2,3]. Soil erosion is the process of moving soil particles by external forces, such as mass movement, wind, and water [4,5]. In Europe, where a humid climate dominates, water-induced soil erosion is the main form of erosion, which poses a serious environmental concern in many European countries [6]. Furthermore, soil erosion by wind and dust storms is one of the challenges in European countries [7,8,9].

Soil erosion by water has numerous environmental impacts. For instance, detaching soil particles from the upper layer of the soil causes a deterioration in agriculture productivity through the loss of organic matter, nutrients, and soil depth [10]. Moreover, moving soil particles over vast distances affects the ecosystem service quality in downstream rivers by increasing the sedimentation and the contamination of aquatic life [11,12]. Since measuring soil erosion at a large scale is difficult, expensive, and time consuming, several models have been developed in recent decades to estimate soil erosion [13,14,15].

In Europe, the Universal Soil Loss Equation (USLE) [15], and its modified version, the Revisited Universal Soil Loss Equation (RUSLE) [14], is the most widely used in quantifying soil erosion at multiple scales across Europe. At large spatial scales, RUSLE is typically the most frequently used model to estimate soil erosion [16]. In the RUSLE model, the average annual soil erosion is calculated by multiplying six factors, including the rainfall erosivity factor (R factor). These factors are slope length (L-factor), soil erodibility (K-factor), slope steepness (S-factor), supporting conservation practices (P-factor), and crop type and management (C-factor). In this sense, rainfall erosivity is considered the most important, as rainfall has a direct impact on detaching and moving the soil particles [15].

Rainfall erosivity is the potential force of raindrops to detach and erode soil particles [17]. As it is one of the main causes of floods and landslides, researchers have highlighted rainfall erosivity as an important indicator to be investigated [18]. The rainfall erosivity factor is calculated using rainfall records with 1–5 min precipitation intervals [19]; however, these records are rarely accessible for long enough in most of the world. As a result, the kinetic energy concept has been widely employed to estimate the rainfall erosivity factor from half-hourly or hourly datasets [20].

To accurately estimate the R factor using the kinetic energy concept, it is necessary to measure both the intensity and the kinetic energy of the rain, but it is highly challenging to achieve this directly since the equipment needed is expensive and measuring the distribution of the rainstorm’s drop sizes is a tedious process [21]. To overcome this, researchers have developed numerous empirical equations that describe the relationship between rainfall intensity and its kinetic energy [22]. To provide a comprehensive review of these equations, Dash et al. [21] compared six of the most universal equations in more detail and provided a deep evaluation of their applicability in calculating the R factor. Alternative methods for calculating the R factor include index techniques, such as the Modified Fournier Index (*MFI*), especially when high-resolution rainfall records (half-hourly or hourly) are not available. The *MFI* is one of the methods suggested by Arnoldus [23] for calculating the R factor based on the monthly rainfall data. However, some adjustment is required for calculating the R factor based on the *MFI* result [24]. The *MFI* was used to estimate catastrophic erosion by evaluating rainfall erosivity and its association with other meteorological factors [25]. Previously, the *MFI* was implemented in many parts of the world, as can be seen in Table 1.

Recently, the artificial neural network (ANN) and machine learning algorithms have been widely used to predict environmental processes (erosion, contamination, and drought) in many parts of the world [29]. For instance, the multilayer perceptron neural network (MLPNN) model is one of the most widely used models for predicting hydrological data [30]. Mishra and Desai [31] used the ANN, RBF, and adaptive neural network-based fuzzy inference system (ANFIS) to forecast drought (SPI) at various timescales and found that ANN has better performance than RBF and ANFIS. In Iran, MLP, ANFIS, and multiple linear regression models were used for forecasting precipitation; the output showed that MLP produced better results [32]. Jalalkamali et al. [33] compared stochastic models with the ANN to forecast SPI-9 in Iran, and their results revealed that stochastic models performed better. The different model results depend on the drought index and its scale [34]. More examples of the implementation of the ANN for predicting certain environmental variables are presented in Table 2.

Based on the literature, few studies used the ANN to predict rainfall erosivity. However, limited information is available on *MFI* changes in Central Europe. Thus, the main goals of this research were to: (1) assess the Modified Fournier Index (*MFI*) as a representative for the erosivity index in three stations in Hungary between 1901 and 2020; (2) evaluate the ability of ANNs (multilayer perceptron (MLP) and the radial basis function (RBF)) to predict the *MFI*; and (3) rate the importance of input variables in predicting the *MFI* based on sensitivity analysis (∂). Overall, the implementation of ANN to predict *MFI* is still less common, which give this work novelty in its field, where the output will serve researchers, planners, and decision makers.

## 2. Materials and Methods

### 2.1. Data Collection

Data were collected from the Hungarian Metrological Center (https://www.met.hu/en/eghajlat/magyarorszag_eghajlata/eghajlati_adatsorok/Pecs/adatok/havi_adatok/, accessed on 1 June 2022). The data included the monthly rainfall (mm), daily maximum precipitation in the month (mm), monthly mean temperature (°C), daily maximum mean temperature in the month (°C), and daily minimum mean temperature in the month (°C) and were collected from three meteorological stations: Budapest (47°30′40′′ N, 19°01′41′′ E), Debrecen (47°29′44′′ N; 21°37′48′′ E), and Pécs (46°04’37′′ N, 18°13’29′′ E). Interestingly, the data cover 120 years from 1 January 1901 to 1 December 2020.

### 2.2. Modified Fournier Index (MFI)

Rainfall erosivity (R) represents the ability of rain drops to initiate erosion. To calculate the R factor, the measurement of rainfall intensity and rainfall duration is required [40]. As such data are not available in many places in the world, many indices were developed to determine the R factor. The Modified Fournier Index (*MFI*), which was proposed by Arnoldus [23], is a widely used index for estimating the R factor. The *MFI* is based on monthly precipitation (*p_i_*) and total yearly precipitation (*P_total_*):

(1) $$MFI=\overset{i=12}{\underset{i=1}{\sum }}\frac{p_{i}^{2}}{P_{total}}$$

The output of Equation (1) can be categorized as presented in Table 3. Based on that, the *MFI* will have high values where the rainfall values are high. In this sense, regions with high amounts of total annual rainfall and rainfall precipitation concentration will have a high *MFI* value [41]. However, a strong correlation between *MFI* and R-factor was recorded in the literature [41,42]. Overall, the calculation of the *MFI* could provide a realistic estimation of the potential rainfall erosivity factor [43].

### 2.3. Predicting Rainfall Erosivity Based on Artificial Neural Network (ANN)

#### 2.3.1. Thermotical Background of ANN Algorithms (MLP and RBF)

The artificial neural network consists of various interconnected neurons, nodes, or perceptrons that are called artificial neurons. Each node transmits a signal to another node; therefore, it can keep the information between various connections and distinguish the patterns [44]. The interconnected node obtains signals, processes them, and transforms them further. The transferring signal between nodes is a real number, and its output can be estimated using a nonlinear function by summing up all the inputs. The output of any network architecture works as an input for the preceding neuron [45,46].

There are several neural networks, but the multilayer perceptron (MLP) is widely used in environmental studies. The MLP connects nodes in a feedforward ANN. The MLP connections between nodes cannot form a cycle. The MLP is sometimes used as any feedforward ANN, and sometimes it refers to a network with various layers [47].

The MLP is a supervised learning technique used in backpropagation for training the dataset and has the ability to split the data that are not linearly separable. These attributes differentiate it from the linear MLP [48]. Ali et al. [49] used the MLP and found that it has the potential to predict drought as one of the ecosystem components in different performance measures. Therefore, in this study, we also used the MLP model. To estimate the *y* using a three-layer network with *n* number of neurons in the hidden layers and *m* number of inputs, we can use Equation (2):

(2) $$y=f\left[\sum_{k=0}^{n}w_{j.g\;\left(\sum_{i=1}^{m}\;W_{ji}\;x_{i}+w_{j0}\right)+w_{0}}\right]$$

Here, weight *w_j_,* joined with the *j*th neuron in the hidden layer and the output layer *w_ji_* weight make a connection between the *i*th input variable and the *j*th neuron in the hidden layer, where *x_i_* is the *i*th independent variable, *w_j_*_0_ is the bias of the *j*th neuron, *g* is the activation function for the neuron of the hidden layer, and *f* is the activation function for the output layer [50]. 

The radial basis function (RBF) is another form of ANN, which was used in this research. The RBF was first proposed in 1988 by Broomhead and Lowe to solve the ill-conditioned problems in interpolation [51]. The RBF is a base of radial networks comprising neural network groups, i.e., a statistical neural network. Euclidean distance is the net input for the activation function of a neuron between its weight (*w*) and vector (*i*) multiplied by the bias *b*. The equation below (Equation (3)) presents the radial basis function network [50,52]:

(3) $$a=\left(𝕝w-i𝕝b\right)$$

Despite the differences between these two algorithms (i.e., MLP and RBF), both were used for predicting the *MFI* values in Central Europe.

#### 2.3.2. Modeling Framework

##### Input Variable

**Figure 1:**
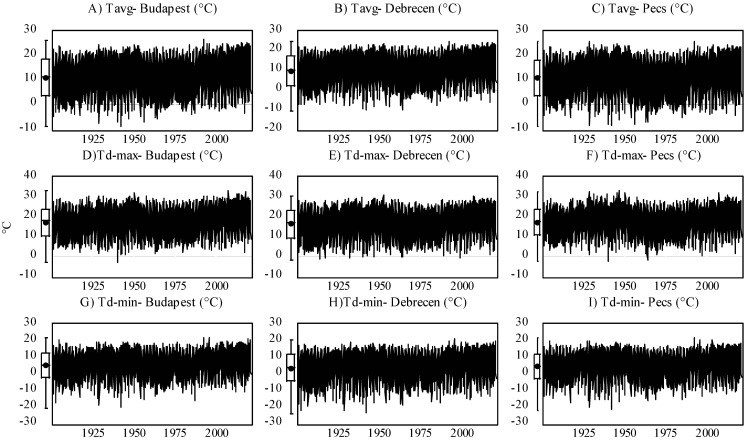
Evolution of monthly mean temperature (*T_avg_*) (**A**–**C**), daily maximum mean temperature (*T_d-max_*) (**D**–**F**), and daily minimum mean temperature (*T_d-min_*) (**G**–**I**) in Central Europe (Budapest, Debrecen, Pécs) between 1901 and 2020.

**Figure 2:**
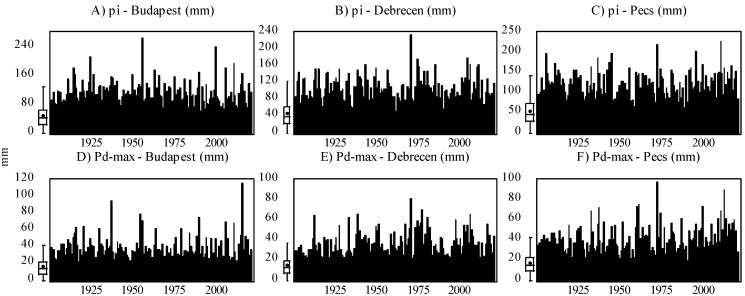
Evolution of monthly rainfall (*p_i_*) (**A**–**C**) and daily maximum precipitation (*P_d-max_*) (**D**–**F**) in Central Europe (Budapest, Debrecen, Pécs) between 1901 and 2020.

Based on Equation (1), the only necessary data for calculating the *MFI* is rainfall data (*p_i_* and *P_total_*). However, for the modeling approach, we engaged other climatic factors, including the daily maximum precipitation (mm) (*P_d-max_*), monthly mean temperature (°C) (*T_avg_*), daily maximum mean temperature (°C) (*T_d-max_*), and daily minimum mean temperature (°C) (*T_d-min_*). An overview of the input variable for each station is presented in Figure 1 and Figure 2 and Table 4.

For the modeling approach, five scenarios were adopted, as can be seen in Table 5. The main purpose of adopting different scenarios is to assess the function of the ANN (MLP and RBF) in predicting the *MFI* based on different input variables. For instance, the first scenario includes all input variables (rainfall (daily + monthly + total) + temperature (monthly)), while the last scenario includes only two rainfall parameters.

##### Training, Testing, and Sensitivity Analysis for Different ANN (MLP and RBF) Algorithms

For the five implemented scenarios and ANN (MLP and RBF) algorithms, data were divided randomly into 70% for training and 30% for testing. As this work was conducted in an SPSS environment, the initial conduction for each algorithm was adopted. For instance, the number of layers of hidden units was from 1 to 50, and the training type was Batch (initial Lambda = 0.000005) for the MLP algorithm, while the architecture of the RBF algorithm was based on automatically finding the number of units in the hidden layer with the normalized RBF as an activation function. 

**Figure 3:**
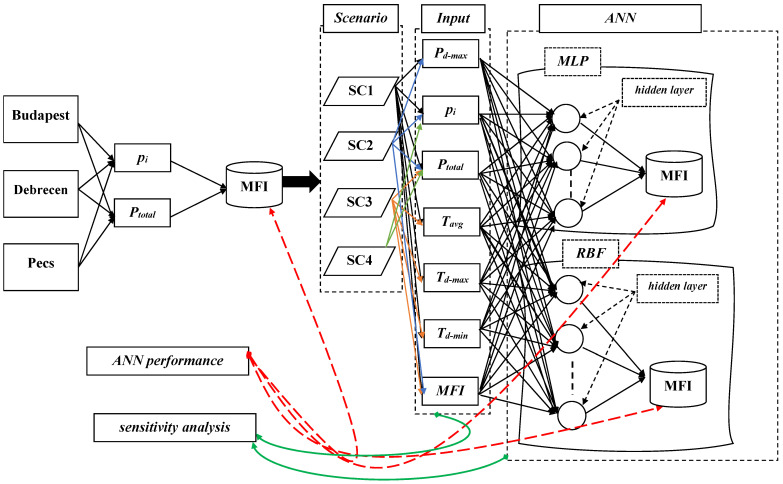
A flowchart depicting the steps adopted in this research: (1) *MFI* calculation, (2) Scenarios, (3) ANN modeling, (4) ANN performance, (5) sensitivity analysis.

Finally, sensitivity analysis (∂) was used to highlight the relationship between the input variable for each scenario and the predicted *MFI*, as shown in Figure 3.

##### Assessing the ANN Performance

To assess the performance of the ANN algorithms (MLP and RBF) in predicting the *MFI*, four indices were used. The indices are model efficiency (*NSE*) [53], index of agreement correlation (*d*) [54], root mean square error (*RMSE*) [55], and Pearson correlation coefficient (*r*) [56], as shown in Table 6.

Additionally, the Taylor diagram [57] was used to plot the $MFI_{Cal}$ against $MFI_{Prd}$. In this sense, the Taylor diagram provides a full overview of the best model/scenarios (Table 5) based on the correlation and standard deviation. 

Finally, it is important to mention that all input and output along with the modeling approach was conducted in IBM SPSS Statistics (V. 24). The SPSS was chosen as it provides a user-friendly platform along with a variety of options that could optimize the output and ANN algorithm. However, we used the initial recommended sets (i.e., batch is the type of training, initial Lambda is 0.0000005, initial Sigma is 0.00005) by SPSS for conducting the modeling.

## 3. Results

### 3.1. MFI Variability in Hungary

**Figure 4:**
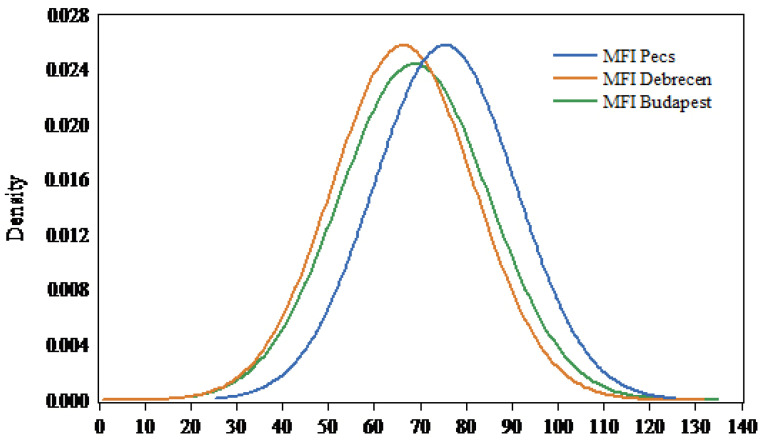
Normal distribution of *MFI* values for studied stations between 1901 and 2020.

In the three studied stations, the *MFI* follows a normal distribution (Figure 4). The average *MFI* values were between 66.30 ± 15.40 (low erosivity) in Debrecen and 75.39 ± 15.39 (low erosivity) in Pecs (Table 7). In Budapest (central Hungary), the highest *MFI* (129.17, high) value was recorded in 1955, which corresponds to 898.8 mm of rainfall, while the lowest *MFI* value (37.21) was recorded in 1997. In Debrecen (eastern Hungary), the highest *MFI* value was 126.23 (high) in 1977. However, the maximum *MFI* value in Pecs (southern Hungary) was 121.09 (high) in 1972. Interestingly, the highest frequency of the *MFI* values was 31 in Budapest, 29 in Debrecen, and 24 in Pecs for the values (65.04, 74.32), (54.28, 63.37), and (81.41, 89.53), respectively (Figure 4). 

### 3.2. MFI Prediction by ANN–MLP and ANN–RBF

**Figure 5:**
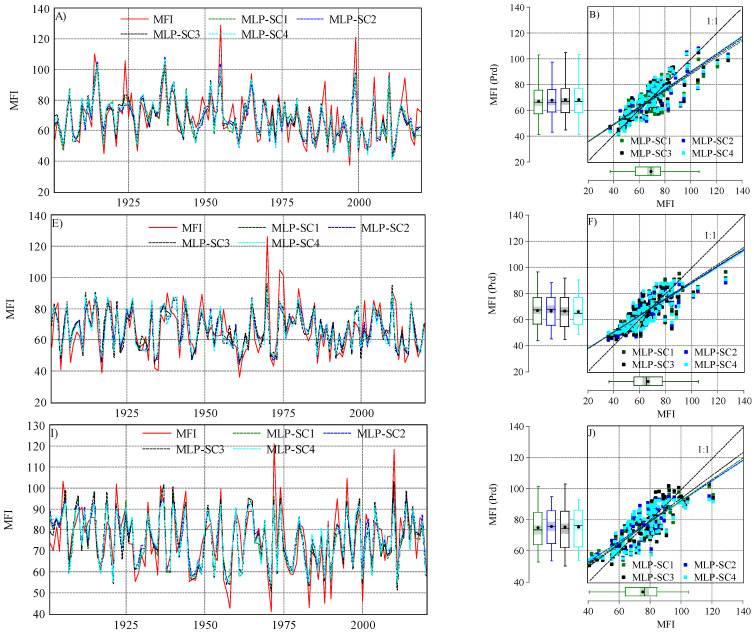
Observed and predicted *MFI* values when using the ANN–MLP algorithm in the studied station under four scenarios (SC1, SC2, SC3, and SC4): (**A**) evolution of observed and predicted *MFI*s in Budapest (1901–2020); (**B**) scatter plot of *MFI_observed_* vs. *MFI_predicted_* in Budapest; (**E**) evolution of observed and predicted *MFI*s in Debrecen (1901–2020); (**F**) scatter plot of *MFI_observed_* vs. *MFI_predicted_* in Debrecen; (**I**) evolution of observed and predicted *MFI* in Pecs (1901–2020); (**J**) scatter plot of *MFI_observed_* vs. *MFI_predicted_* in Pecs.

**Figure 6:**
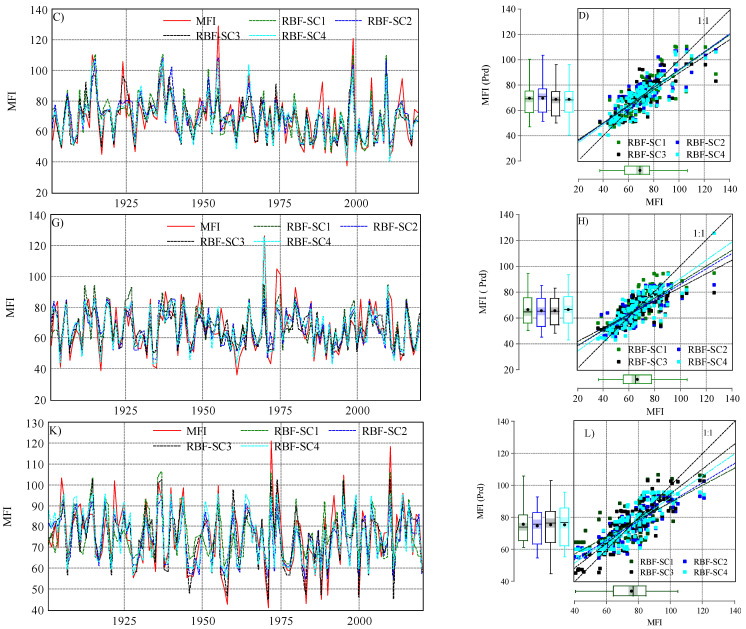
The output of ANN–RBF algorithm (predicted *MFI*) and observed one in the studied station under four scenarios (SC1, SC2, SC3, and SC4): (**C**) evolution of observed and predicted *MFI* in Budapest (1901–2020); (**D**) scatter plot *MFI_observed_* vs. *MFI_predicted_* in Budapest; (**G**) evolution of observed and predicted *MFI* in Debrecen (1901–2020); (**H**) scatter plot *MFI_calculated_* vs. *MFI_predicted_* in Debrecen; (**K**) evolution of observed and predicted *MFI* in Pecs (1901–2020); (**L**) scatter plot *MFI_calculated_* vs. *MFI_predicted_* in Pecs.

In the three stations, a combination of different climate variables (four scenarios) was used by ANN–MLP and ANN–RBF for predicting the *MFI* values. The predicted *MFI* values are presented in Figure 5 and Figure 6.

**Figure 7:**
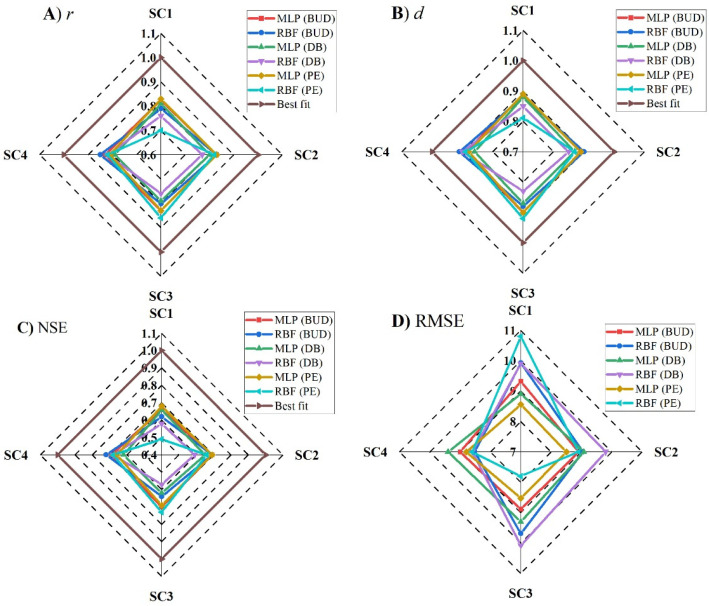
Performance analysis of ANN (MLP and RBF) algorithms in predicting *MFI* values under four scenarios (SC1, SC2, SC3, and SC4) at three stations in Budapest (BUD), Debrecen (DB), and Pecs (PE): (**A**) Pearson correlation coefficient (*r*), (**B**) index of agreement correlation (*d*), (**C**) model efficiency (*NSE*), and (**D**) root mean square error (*RMSE*).

For the ANN–MLP, each scenario exhibited a different performance in predicting *MFI* values (Figure 5). In Budapest, the Pearson correlation coefficient (*r*) ranged between 0.82 (SC1-MLP) and *r*_*MFI* vs. *MFI prd*_ = 0.83 for the rest of the scenarios. The *d* index ranged between 0.88 (SC1-MLP) and 0.9 (SC3-MLP). The efficiency of the ANN–MLP was assessed using the *NSE*. However, the *NSE* value was above 0.6, which indicates a good model performance for all scenarios. However, the highest value was *NSE* = 0.7 in SC3. Interestingly, the highest *NSE* value and lowest *RMSE* were recorded in SC3. Based on the statistical indicator, the efficiency of the scenarios in predicting the *MFI* can be highlighted as follows: SC3 > SC2 > SC4 > SC1. For Debrecen, the ANN–MLP exhibited a good performance (Figure 7). The *r* values and those of other statistical indicators were lower than those recorded in Budapest. For instance, the *r* ranged between 0.79 and 0.81, and the *NSE* between 0.62 and 0.66, while the *RMSE* was higher than Budapest. Based on the four suggested scenarios, the ANN–MLP performance could be ranked as follows: SC1 > SC2 > SC4 > SC3. Similar to Budapest, the ANN–MLP performance in Pecs was better than that in Debrecen (Figure 7). The *d* index was higher than 0.88, and the *NSE* was good (*NSE* > 0.66). Based on this, we can draw the following rank: SC2 > SC3 > SC1 > SC4.

Similar to ANN–MLP, the ANN–RBF showed a good ability to predict the *MFI* under different scenarios (Figure 6). In Budapest and Debrecen, SC4 had the highest correlation *r_MFI *vs.* MFI prd_* (0.85, 0.82), with the highest and lowest *NSE*, respectively, which indicates that SC4 (ANN–RBF) (*p_i_* + *P_total_*) is the best scenario for Budapest and Debrecen. In this sense, the scenarios can be ranked for both stations as follows: SC4 > SC2 > SC3 > SC1 (Figure 7). In Pecs, SC3 (ANN–RBF) (*P_total_* + *T_avg_* + *T_d-max_* + *T_d-min_*) outperformed the rest of the scenarios (*r_MFI *vs.* MFI prd_* = 0.86, *d* = 0.92, *NSE* = 0.73, *RMSE* = 7.8). However, the performance of the four scenarios can be ranked as SC3 > SC4 > SC2 > SC1.

**Figure 8:**
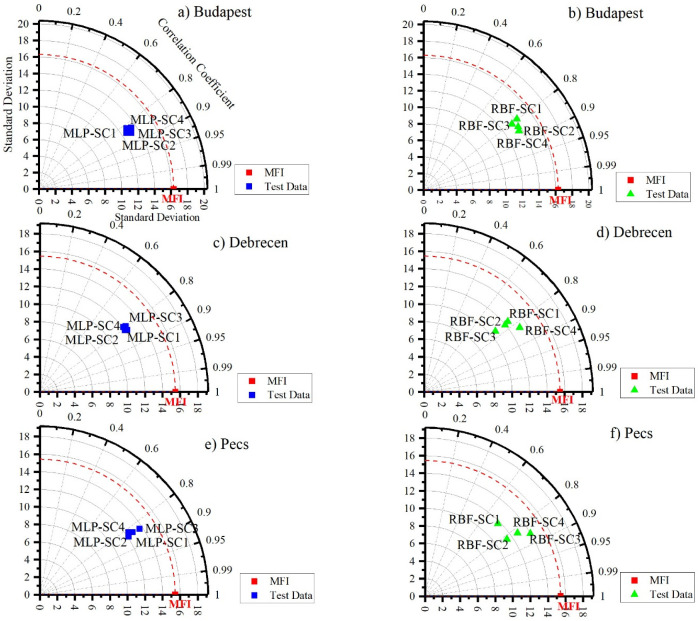
Taylor diagram for assessing the performance of ANN (MLP and RBF) algorithms in predicting *MFI*. (**a**,**b**) Budapest. (**c**,**d**) Debrecen. (**e**,**f**) Pecs.

The Taylor diagram (Figure 8) reveals that SC2 and SC3 for Budapest and SC1 and SC2 for Debrecen and Pecs are the best scenarios in terms of the ANN–MLP (Figure 8). However, for the ANN–RBF, SC4 was the most appropriate scenario for Budapest and Debrecen, while SC3 was the best one for Pecs. Overall, these analyses promoted SC2 (*P_d-max_* + *p_i_ + P_total_*) and SC4 *(P_total_* + *T_avg_* + *T_d-max_* + *T_d-min_)* as the best scenarios for predicting *MFI* using the ANN–MLP and ANN–RBF, respectively.

### 3.3. Comparing between ANN–MLP and ANN–RBF in MFI Prediction

**Figure 9:**
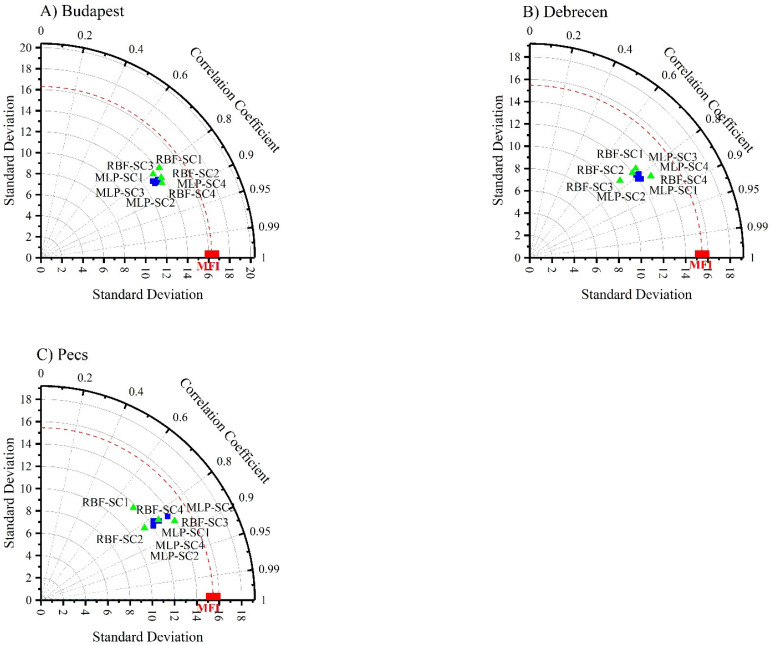
Analysis of ANN–MLP and ANN–MLP RBF performance in predicting *MFI* using a Taylor diagram. (**A**) Budapest. (**B**) Debrecen. (**C**) Pecs.

To compare the outputs of each algorithm in each station, the outputs were plotted in a Taylor diagram (Figure 9). The main point of this step is to test all the scenarios for both algorithms against the calculated MF. For Budapest and Debrecen stations, the RBF-SC4 followed by the MLP-SC2 was the best predictor. In Pecs, the RBF-SC3 followed by the MLP-SC1 was superior compared to the others. Notably, in the three stations, the RBF-SC1 had the worst performance (Figure 9). Interestingly, the RBF outperformed the MLP. 

### 3.4. Independent Variable Importance and Sensitivity Analysis

**Figure 10:**
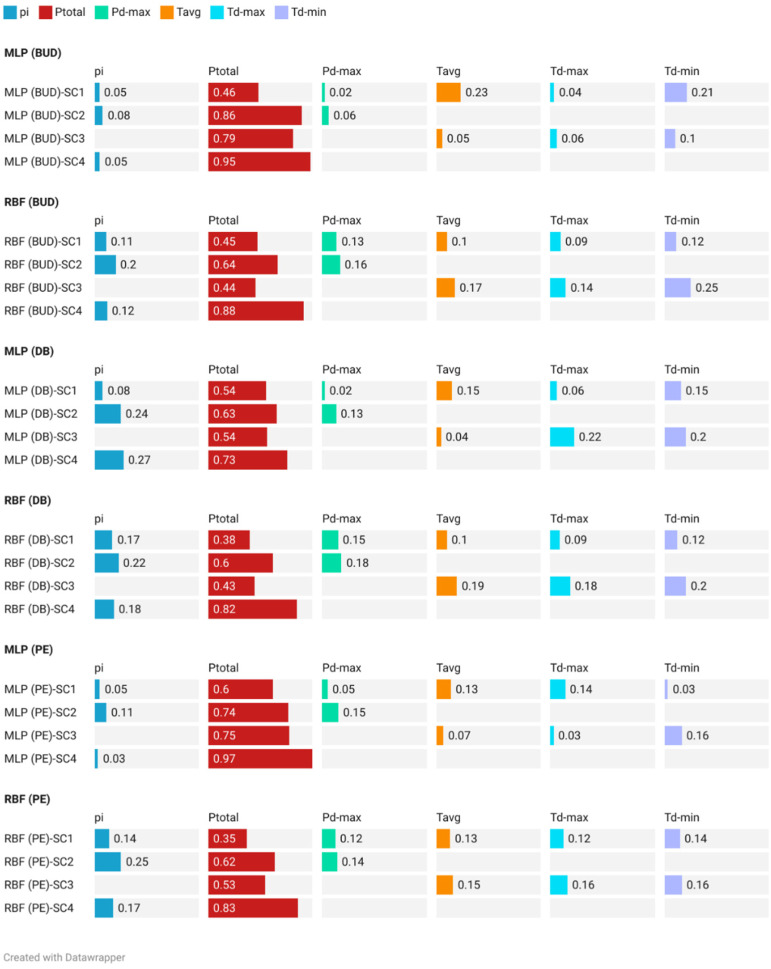
Sensitivity analysis for ANN (MLP and RBF) algorithms in the studied station under four scenarios.

The main goal of sensitivity analysis is to highlight the importance of the input variables in the prediction process. For the MLP in the Budapest station, the *P_total_* had the highest importance in all the suggested scenarios (∂_SC1_ = 0.46; ∂_SC2_ = 0.86; ∂_SC3_ = 0.79; ∂_SC4_ = 0.95), followed by *T_avg_* in SC1, and *T_d-min_* in SC3 (Figure 10). For the RBF in the same station, the *P_total_* also had the highest importance (∂_SC1_ = 0.45; ∂_SC2_ = 0.64; ∂_SC3_ = 0.44; ∂_SC4_ = 0.88). However, other independent variables exhibited a good level of importance. For example, in SC1 *T_d-min_*, *T_d-max_* and *p_i_* showed ∂_SC1_ importance ranging between 0.13 and 0.11, while in SC2, the *p_i_* importance reached ∂_SC2_ = 0.2 (Figure 10).

For the second station (Debrecen), both algorithms showed that *P_total_* has an important role in *MFI* prediction. In the MLP, the importance value reached ∂_SC4_ = 0.73, while it was ∂_SC4_ = 0.82 in the RBF. Notably, the next important variable was the *p_i_*, with ∂_SC2-MLP_ = 0.24 and ∂_SC2-MLP_ = 0.22. At the Pecs station, the importance of the *P_total_* was more pronounced for both the MLP (∂_SC4_ = 0.97) and RBF(∂_SC4_ = 0.83) (Figure 10).

Based on the four scenarios and both ANN (MLP and RBF) algorithms, the sensitivity analysis showed that *P_total_*, *p_i_*, and *T_d-min_* had the highest relative importance in the prediction process. 

## 4. Discussion

In this research, the *MFI* was calculated for tracking rainfall erosivity in Central Europe; then, two ANN (RBF and MLP) algorithms were tested to assess their ability in the prediction of the *MFI*. At the three studied stations, the *MFI* values ranged from very low to high (1901–2020) (Table 7). Previously, De Luis et al. [41] analyzed the erosivity trend in Western Europe (Iberian Peninsula) and detected a notable decrease in rainfall erosivity based on the *MFI* (1951–2000). For the Netherlands, Lukić et al. [10] reported that the *MFI* values ranged between 77.93 and 97.27 (1957–2016). However, changes in erosivity class from low to moderate were reported in the same study. These changes in rainfall erosivity in Europe can be mainly explained by climate change (i.e., extreme events: flood and drought), which largely affects the precipitation patterns, not only in Europe but all over the world [58,59,60,61].

The output of RBF and MLP showed that the RBF outperformed the MLP. However, both algorithms were perfectly capable of predicting the *MFI* values, with some differences. The differences between the output could be explained by the way that each algorithm works. The necessary step for the proper functioning of the NN is to optimize the weights, known as calibration. Different types of algorithms can be used to optimize the weight, e.g., back propagation [62] and Levenberg–Marquardt [63]. These algorithms can minimize the disparity between forecasted and observed values by adjusting the network weight [46].

Generally, the ANN works on the principle of the training dataset. There are various kinds of neural network (NN) models, but usually, two models are used in prediction applications, i.e., recurrent network and feedforward network. The backpropagation algorithm is used to train both models [49,50,51,52,53,54,55,56,57,58,59,60,61,62,63,64]. When the backpropagation algorithm is used to change the weight of neurons, it works on the gradient descent method (weights change in downward direction). The signal strength between nodes is directly dependent on the weights of neurons [49]. Feedforward NN is a basic type, and it is capable of estimating constant and integral functions.

The network architecture of MLP comprises neurons put together into layers. The MLP contains three layers of nodes, i.e., input, hidden, and output layers. The MLP can have one or more hidden layers with various numbers of neurons. In addition to the input node, the hidden and output nodes are considered neurons [65]. When we used the MLP to study rainfall erosivity (*MFI*), the input layer contained the variables (*P_d-max_*, *p_i_*, *P_total_*, *T_avg_*, *T_d-max_*, and *T_d-min_*), and the output layer presented the predicted *MFI* (Figure 3), while the hidden layer included a nonlinear function and utilized weight for the input layer. Neurons in the hidden layer work in a trial and error approach [34].

The MLP and RBF consist of three network layers; however, the main difference between the RBF and MLP is that the RBF’s hidden and output layers are different, unlike those of the MLP [66]. The hidden layer neurons are nonlinear, while the output layer neurons are linear in the RBFs. The nonlinear hidden layer neuron plays a significant role in the nonlinear modeling task [67]. The RBF network is simpler compared to MLP. However, the MLP is more successfully implemented in various complex problems. The RBF is a local approximation network, and its output can be estimated by hidden units in a local receptive field. The MLP network works globally, and its output is determined by all the neurons [68]. Despite the similarity between both algorithms, the differences in the architecture process led to different output and accuracy (Figure 7, Figure 8 and Figure 9).

Overall, the implementation of the ANN for predicting the *MFI* or other hydrological and environmental variables was proven to be a useful tool for predicting and forecasting [69]. However, the output of this research could be useful for local planners on a county scale for predicting the *MFI* values based only on monthly and yearly rainfall.

## 5. Conclusions

Land degradation is a major issue all over the world due to its negative impact on the agroecosystem and environmental components. Recently, machine learning and the artificial neural network have been implemented in environmental research for predicting natural hazards. In this research, ANN (MLP and RBF) algorithms were implemented to predict the *MFI* as a representative of erosivity factor (soil erosion) in Central Europe. Five scenarios with different inputs (rainfall and temperature) were suggested for exploring the accuracy of ANN (MLP and RBF) algorithms. The output of this research can be summarized as follows:

1- The *MFI* ranged between 91.97 (Budapest) and 80.25 (Pecs), with a notable decrease in *MFI* values (1901–2020).
2- The SC2 (*P_d-max_* + *p_i_ + P_total_*) was the best scenario for predicting the *MFI* using the ANN–MLP.
3- The SC4 *(P_total_* + *T_avg_* + *T_d-max_* + *T_d-min_)* was the most accurate scenario for predicting the *MFI* by using the ANN–RBF.
4- The sensitivity analysis revealed that *p_i_* followed by *P_total_* are the most important input variables for predicting *MFI* values.

It is good to mention that this research was only focused on *MFI* as one of the factors that contribute to soil erosion based on the monthly rainfall data. Some other factors such as land use (agricultural areas), soil properties (i.e., texture, structure), vegetation cover, and inclination angle of rainfall streams were not considered in this research.

Local planers, environmental organizations, and decision makers will be able to use the output of this research, where the prediction of the *MFI* could be performed to a satisfactory level based on the total rainfall in the target regions. In the next steps, other machine learning methods will be implemented to test their accuracy in the prediction of the *MFI*. However, the output of this research could serve as a good result for both scientific and industrial communities.

## Figures and Tables

**Table 1:** Some examples of implementation of *MFI* in different parts of the world.

Country	Period	Comment	Reference
NE Spain	1997–2006	*MFI* has poor performance against observed R-value	Angulo-Martínez and Beguería [24]
India	1981–2019 (CHIRPS-2.0 Global_daily)	High correlation between *MFI* and R	Raj et al. [26]
Uruguay	1931–2000	*MFI* increased in the 1980s and 1990s	Munka et al. [27]
Iran	1970–1992	Roose’s index more efficient than *MFI*	Sadeghi [28]
Pannonian basin (Central Europe)	1961–2014	Low erosive class of *MFI* was recorded	Lukić et al. [11]

**Table 2:** Implementation of ANN for predicting some environmental variables in many parts of the world.

Country	Environmental Phenomenon	Method	Comment	Reference
Two watersheds in Australia and France	Rainfall-runoff modeling	EANN and FFNN	EANN is better than FFNN	Nourani [35]
Kasilian watershed (Iran)	Soil erosion	ANN and GIS	ANN with GIS perfectly predicts soil erosion	Gholami et al. [36]
Langat River basin (Malaysia)	Meteorological drought	ANN-ARIMA	Engagement of Wavelet-based ARIMA–ANN was recommended	Khan et al. [37]
Bojnourd (Iran)	Drought forecasting	ANN, ANFIS, support vector machine (SVM)	SVM was the most accurate one	Mokhtarzad et al. [38]
South Korea	Groundwater level	ANN and SVM	SVM was better than ANN	Yoon et al. [39]

**Table 3:** Description of the *MFI* erosivity range [42].

*MFI* Value	Category
<60	Very low
60–90	Low
90–120	Moderate
120–160	High
>160	Very high

**Table 4:** Descriptive statistical analysis of input variables in three locations, Budapest, Debrecen, and Pécs, between 1901 and 2020.

Location	Statistic	n *	Min.	Max.	Range	Median	X	SD	SK	KU
Budapest	*P_d-max_*	1440	0.0	115.4	115.4	14.2	16.6	11.5	1.8	7.2
	*p_i_*	1440	0.0	263.1	263.1	42.8	48.5	33.4	1.3	2.9
	*T_avg_*	1440	−8.7	26.6	35.3	11.5	11.3	8.0	−0.1	−1.2
	*T_d-max_*	1440	−2.9	33.1	36.0	18.1	17.4	7.7	−0.2	−1.2
	*T_d-min_*	1440	−20.6	21.7	42.3	5.5	5.1	8.5	−0.2	−1.0
Debrecen	*P_d-max_*	1440	0.0	80.3	80.3	13.0	15.4	10.7	1.7	4.2
	*p_i_*	1440	0.0	232.3	232.3	40.3	47.2	32.0	1.2	1.8
	*T_avg_*	1440	−10.2	24.9	35.1	10.4	10.0	8.3	−0.2	−1.2
	*T_d-max_*	1440	−1.6	30.7	32.3	17.4	16.4	7.8	−0.2	−1.1
	*T_d-min_*	1440	−23.9	20.8	44.7	3.9	3.5	9.3	−0.3	−0.9
Pécs	*P_d-max_*	1440	0.0	97.4	97.4	15.4	18.0	11.8	1.7	4.6
	*p_i_*	1440	0.0	227.0	227.0	47.7	55.0	35.5	1.1	1.3
	*T_avg_*	1440	−8.6	25.9	34.5	11.5	10.9	8.0	−0.2	−1.2
	*T_d-max_*	1440	−2.3	32.8	35.1	17.9	17.3	7.4	−0.2	−1.1
	*T_d-min_*	1440	−21.8	21.7	43.5	4.9	4.4	8.6	−0.3	−0.9

* *n*: number of observations; Min.: minimum; Max.: maximum; X: mean; SD: standard deviation (*n*); SK: skewness (Pearson); KU: kurtosis (Pearson).

**Table 5:** Developed scenarios for *MFI* prediction based on ANN (MLP and RBF) in Central Europe.

Number	Scenarios	Input	Climate Element
1	SC1	*P_d-max_* + *p_i_ + P_total_ + T_avg_* + *T_d-max_* + *T_d-min_*	Rainfall (daily + monthly + total) + temperature (monthly)
2	SC2	*P_d-max_* + *p_i_ + P_total_*	Rainfall (daily + monthly + total)
3	SC3	*P_total_* + *T_avg_* + *T_d-max_* + *T_d-min_*	Rainfall (total) + temperature (monthly)
4	SC4	*p_i_ + P_total_*	Rainfall (monthly + total)

**Table 6:** Indices for evaluation of ANN performance for predicting *MFI* erosivity.

Index	Equation *	Range	Note
*NSE*	$NSE=1-\frac{\sum_{i=1}^{n}{\left(MFI_{Prd}-MFI_{Cal}\right)}^{2}}{\sum_{t=1}^{n}{\left(MFI_{Cal}-\bar{MFI_{Cal}}\right)}^{2}}$	−∞ and 1	$\mathrm{When}\;\mathrm{the}\;\mathrm{NSE}\;\mathrm{reaches}\;1,\;\mathrm{it}\;\mathrm{is}\;a\;\mathrm{perfect}\;\mathrm{match}\;\mathrm{between}\;\;MFI_{Cal}$ $\;\mathrm{and}\;MFI_{Prd}$
*d*	$d=1-\frac{\sum_{i=1}^{n}{\left(MFI_{Cal}-MFI_{Prd}\right)}^{2}}{\sum_{i=1}^{n}{\left(\left|MFI_{Prd}-\bar{MFI_{Cal}}\right|+\left|MFI_{Cal}-\bar{MFI_{Cal}}\right|\right)}^{2}}$	0 to 1	$\mathrm{When}\;d\;\mathrm{approaches}+1,\;\mathrm{this}\;\mathrm{indicates}\;\mathrm{an}\;\mathrm{ideal}\;\mathrm{agreement}\;\mathrm{between}\;\;MFI_{Cal}$ $\;\mathrm{and}\;MFI_{Prd}$
*RMSE*	$RMSE=\sqrt{\frac{\sum_{i=1}^{n}{\left(MFI_{Cal}-MFI_{Prd}\right)}^{2}}{n}}$	0 to +∞	$A\;\mathrm{lower}\;\mathrm{RMSE}\;\mathrm{value}\;\mathrm{denotes}\;a\;\mathrm{perfect}\;\mathrm{match}\;\mathrm{between}\;\;MFI_{Cal}$ $\;\mathrm{and}\;MFI_{Prd}$
*r*	$r=\left[\frac{\sum_{i=1}^{n}\left\{\left(MFI_{Cal}-\bar{MFI_{Cal}}\right)\left(MFI_{Prd}-\bar{MFI_{Prd}}\right)\;\right\}}{\sqrt{\sum_{i=1}^{n}{\left(MFI_{Cal}-\bar{MFI_{Cal}}\right)}^{2}}\sqrt{\sum_{i=1}^{n}{\left(MFI_{Prd}-\bar{MFI_{Prd}}\right)}^{2}}}\right]$	−1 to +1	$\mathrm{When}\;r=+1,\;\mathrm{this}\;\mathrm{shows}\;\mathrm{an}\;\mathrm{ideal}\;\mathrm{positive}\;\mathrm{linear}\;\mathrm{relationship}\;\mathrm{between}\;\;MFI_{Cal}$ $\;\mathrm{and}\;MFI_{Prd}$

*$MFI_{Cal}$: calculated *MFI* value based on Equation (1); $MFI_{Prd}$: predicted value based on the ANN algorithms (MLP and RBF); $\bar{MFI_{Cal}}$: average of calculated values; $\bar{MFI_{Prd}}$: average of predicted values.

**Table 7:** An overview of *MFI* values in the three studied stations (1901–2020).

Station	n *	Min	Max	Range	MD	Mean	SD	SK	K
Budapest	120	37.21	129.17	91.97	68.66	68.72	16.24	0.90	1.43
Debrecen	120	36.10	126.23	90.13	64.91	66.30	15.40	0.59	0.76
Pecs	120	40.83	121.09	80.25	76.48	75.39	15.39	0.14	0.09

* *n*: number of observations; Min: minimum; Max: maximum; MD: median; SD: standard deviation (*n*); SK: skewness (Pearson); K: kurtosis (Pearson).

## Data Availability

Data are freely available at: https://www.met.hu/en/eghajlat/magyarorszag_eghajlata/eghajlati_adatsorok/Pecs/adatok/havi_adatok/ (accessed on 1 June 2022).
